# Contribution of two-component regulatory systems to the acute-to-chronic infection transition of *Pseudomonas aeruginosa* in cystic fibrosis

**DOI:** 10.1128/jb.00471-25

**Published:** 2026-02-25

**Authors:** Verónica Roxana Flores-Vega, Gabriela Hernández-Martínez, Miguel Cocotl-Yañez, Miguel A. Ares, Nilton Lincopan, Vianney Ortiz-Navarrete, Roberto Rosales-Reyes

**Affiliations:** 1 Unidad de Medicina Experimental, Facultad de Medicina, Universidad Nacional Autónoma de México 7180, Mexico City, Mexico; 2 Departamento de Biomedicina Molecular, Centro de Investigación y de Estudios Avanzados del IPN 42576 , Mexico City, Mexico; 3 Departamento de Microbiología y Parasitología, Facultad de Medicina, Universidad Nacional Autónoma de México 7180, Mexico City, Mexico; 4 Unidad de Investigación Médica en Enfermedades Infecciosas y Parasitarias, Hospital de Pediatría, Centro Médico Nacional Siglo XXI, Instituto Mexicano del Seguro Social 37767, Mexico City, Mexico; 5 Department of Microbiology, Institute of Biomedical Sciences, University of São Paulo 525539, São Paulo, Brazil; 6 Department of Clinical Analysis, School of Pharmacy, University of São Paulo 534851 , São Paulo, Brazil; Dartmouth College Geisel School of Medicine, Hanover, New Hampshire, USA

**Keywords:** *Pseudomonas aeruginosa*, virulence factors, two-component system, cell death

## Abstract

Cystic fibrosis (CF) is a life-threatening genetic disorder that causes severe dysfunction in the lungs, digestive system, and other organs. Chronic respiratory infections are a significant cause of morbidity and mortality among CF-related complications. *Pseudomonas aeruginosa* is an opportunistic bacterial pathogen and a predominant colonizer of the CF lung. It drives a progressive decline in pulmonary function. This pathogen shows remarkable adaptability. It can establish both acute and chronic infections despite the host’s immune defenses and antimicrobial treatments. *P. aeruginosa* expresses a diverse set of virulence factors, including adhesins, proteases, exotoxins, siderophores, secretion systems, and exopolysaccharides. These factors facilitate host colonization, immune evasion, and disease progression. The transition from acute to chronic infection is mediated by bacterial sensing of environmental signals. These signals dynamically modulate the expression of virulence factors. Also, the hostile conditions within the CF lung drive the acquisition and accumulation of adaptive mutations in the bacterial genome. These genetic modifications often impair the efficiency of DNA repair mechanisms, leading to a hypermutable phenotype. This accelerates bacterial evolution. As a result, acute-phase virulence factors are downregulated, while antimicrobial resistance and biofilm formation increase. This contributes to persistent and refractory infections. In this review, we examine the crucial roles of two-component regulatory systems. These systems fine-tune gene expression, enhance bacterial survival, and regulate the shift from acute to chronic infection. Understanding these adaptive mechanisms is essential for developing novel therapeutic strategies to combat this highly resilient pathogen.

## INTRODUCTION

Cystic fibrosis (CF) is an autosomal recessive genetic disorder caused by mutations in the cystic fibrosis transmembrane conductance regulator (*CFTR*) gene. This gene encodes a transmembrane ion channel that conducts chloride (Cl^−^), sodium (Na^+^), and bicarbonate (HCO_3_^−^) ions across epithelial cells (1, 2). CFTR is expressed in the airways, gastrointestinal and reproductive tracts, pancreas, and sweat glands (3). In airways, the absence or dysfunction of CFTR results in reduced Cl^−^ secretion and increased Na^+^ absorption. These changes lead to ionic imbalances. As a result, thick, sticky, dehydrated mucus accumulates in the lower airways (4), facilitating bacterial colonization (4–6), and pulmonary exacerbations. Ultimately, these processes compromise the patient’s life (7, 8).

Chronic bacterial infection triggers a sustained inflammatory response. This response progressively impairs lung function (9). In individuals with CF (iwCF), persistent inflammation resulting from recurrent infections leads to age-dependent shifts in the respiratory microbiota. The most common bacterial pathogens include *Pseudomonas aeruginosa*, *Staphylococcus aureus*, *Haemophilus influenzae*, and members of the *Burkholderia cepacia* complex. Less frequent pathogens, such as *Stenotrophomonas maltophilia*, methicillin-resistant *S. aureus*, *Mycobacterium abscessus*, *Achromobacter* spp., *Streptococcus milleri*/*anginosus* group, and *Aspergillus fumigatus*, may also be detected (10, 11). Culture-independent methods have revealed additional diversity in the microbiota. Taxa such as *Actinomyces odontolyticus*, *Atopobium parvulum*, *Granulicatella adiacens*, *Gemella haemolysans*, *Fusobacterium* spp., *Mycobacterium* 956, *Neisseria* spp., *Porphyromonas* spp., *Prevotella* spp., *Rothia mucilaginosa*, *Serratia marcescens*, *Streptococcus* spp., and *Veillonella* spp. were also identified in the CF lung microbiome (12, 13).

Chronic bacterial colonization drives exacerbations in iwCF (14). Antimicrobial therapy reduces colonization (15). However, the continued use of antibiotics imposes intense selective pressure. This pressure rapidly promotes the emergence of antibiotic-resistant bacterial variants, compromising treatment efficacy (16). In this context, the introduction of highly effective modulator therapy reduces symptom burden, improves clinical outcomes, and enhances the quality of life for iwCF (17, 18). Notably, the triple combination of elexacaftor, tezacaftor, and ivacaftor has demonstrated superior efficacy in improving the quality of life in individuals with the F508del mutation. Nevertheless, despite these therapies, chronic infections caused by opportunistic pathogens, such as *P. aeruginosa*, persist and continue to pose significant clinical and biological challenges (19–21).

## P. AERUGINOSA

*P. aeruginosa* is a ubiquitous gram-negative bacterium. It has remarkable adaptability to diverse environmental conditions (22). Its large genome ranges from 5.2 to 7 Mbp (23) and enables genomic, phenotypic, and metabolic plasticity. These features support adaptability and survival in diverse environments (24). Adaptability requires detecting environmental cues via quorum sensing (QS) and several two-component systems (TCSs) (25, 26).

The QS is a sophisticated communication system. In this system, bacteria use chemical signals to coordinate group behaviors in response to changes in population density. In *P. aeruginosa*, the QS system consists of four interconnected regulatory networks: (i) Las (elastase), (ii) rhamnolipid (Rhl), (iii) *Pseudomonas* quinolone signal (PQS), and (iv) integrative quorum sensing (IQS). Each network produces distinct signaling molecules that engage their specific receptors. This leads to the activation of gene expression programs linked to persistence and virulence (27, 28).

Furthermore, TCSs are complex regulatory networks. In these, a sensor histidine kinase (HK) detects environmental cues, while a cognate response regulator (RR) modulates the expression of virulence factors (29). *P. aeruginosa* encodes many of these systems, 55 HKs, and 89 RRs (30). The deletion of individual HKs in several TCSs has demonstrated their critical role in virulence regulation (31). Together, these systems detect environmental cues and regulate gene expression associated with adaptation and virulence modulation (32). Consequently, *P. aeruginosa* can cause both acute and chronic infections in iwCF (33).

The transition from acute to chronic infection requires the accumulation of mutations in the bacterial genome during exposure to environmental stressors (34–36). As a result, this process accelerates bacterial evolution and alters the expression of virulence determinants (37, 38). Hallmarks of chronic infection include the production of mucoid variants, loss of pigment production (e.g., pyoverdine and pyocyanin), and reduced secretion of proteases via the type II secretion system. Additionally, there is a reduction in exotoxin secretion (ExoS, ExoT, ExoU, and ExoY) via the T3SS (33, 39–43). At the same time, *P. aeruginosa* enhances interbacterial competition via the type VI secretion system (T6SS) (44). It also enhances alginate production and biofilm formation, thereby increasing antimicrobial tolerance (33, 39, 45).

## MUCUS COMPOSITION IN CF

Airway mucus is composed primarily of water (90%–95%), mucins and other proteins (1%–5%), lipids (1%–2%), and electrolytes (~1%) (46). Mucins are produced and secreted by goblet cells (47). In the airways, goblet cells secrete MUC5AC and MUC5B, whereas submucosal glands predominantly secrete MUC5B. These mucins assemble into filamentous networks that facilitate mucociliary clearance by sweeping the large airways and removing inhaled bacterial pathogens (48).

In CF, mucus composition and properties vary with patient age, bacterial infection status, immune responses, and medical treatments, including aerosolized antibiotics, dornase alfa administration, and other therapies. Abnormal mucus structural organization alters mucin secretion rates, post-secretory expansion, and composition, thereby compromising the protective function of the mucus layer. Mutations in CFTR impair the transport of Cl⁻ and HCO_3_⁻ (49). HCO_3_⁻ is essential for proper mucin hydration and fluidity and for chelating Ca²^+^, a process required for Ca^2+^-Na^+^ exchange that promotes mucin expansion (4). In CF, reduced HCO_3_⁻ secretion increases Ca²^+^ availability, resulting in decreased mucus pH (2.9–6.5) (50, 51), reduced hydration and fluidity, impaired bacterial clearance, and the accumulation of thick, sticky mucus that favors bacterial colonization.

Mucus composition is further modified upon neutrophil recruitment to sites of infection. During immune activation, neutrophils can release neutrophil extracellular traps (NETs), which restrict bacterial dissemination (52). NETs contain calprotectin, the most abundant cytosolic protein in neutrophils, a heterodimer composed of S100A8 and S100A9 (53) that accounts for approximately 40%–60% of neutrophil cytosolic proteins. Calprotectin binds Zn^2+^ and Mn^2+^ with high affinity (54); in contrast, Mg^2+^ with low affinity, thereby reducing its availability in the lung mucus environment. In addition, extracellular DNA released during NET formation is highly anionic and can chelate divalent cations, including Mn^2+^, Ca^2+^, and Mg^2+^ (55). Notably, bacterial biofilms are complex structures whose extracellular matrix also contains extracellular DNA, further modulating local metal availability and mucus physicochemical properties (56).

## ROLE OF THE TWO-COMPONENT SYSTEMS IN THE TRANSITION FROM ACUTE-TO-CHRONIC INFECTION

**Fig 1:**
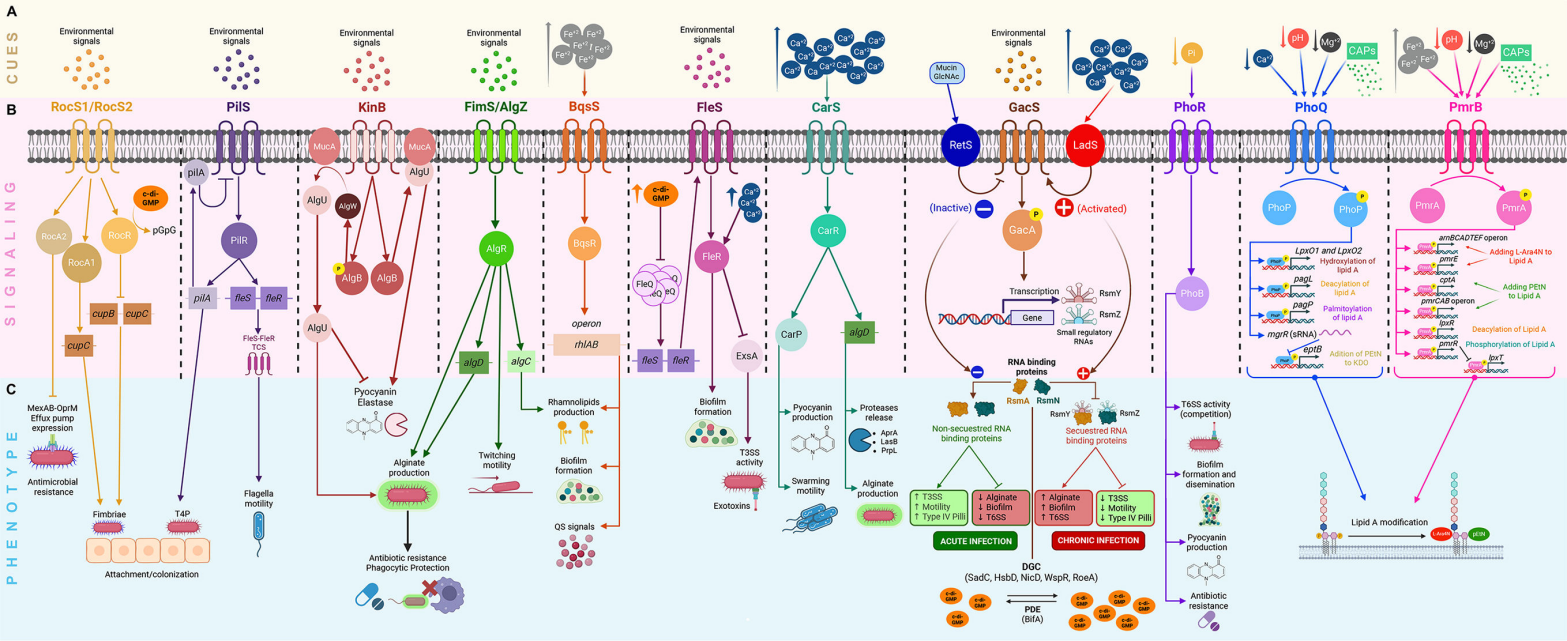
Two-component systems (TCSs) sense environmental signals in the lung during *Pseudomonas aeruginosa* infection. (**A**) Environmental cues present in cystic fibrosis (CF) mucus, including Mg²^+^, Ca²^+^, Fe²^+^, inorganic phosphate (Pi), acidic pH, cationic antimicrobial peptides (CAPs), mucins, and N-acetylglucosamine (GlcNAc), are shown on a yellow background. (**B**) TCSs involved in signal detection and transduction, as well as associated regulatory molecules that mediate transcriptional control of virulence-associated genes, are shown on a pink background. (**C**) Phenotypes resulting from TCS activation are shown on a blue background. RocS1-RocR/RocA1 induces expression of Cup fimbriae, while PilS-PilR regulates type IV pilus (T4P) production, which contributes to surface attachment and can promote activation of the FleS-FleR system, supporting flagellar motility. As *P. aeruginosa* adapts to the lung environment, additional TCSs modulate virulence-associated phenotypes. KinB-AlgB regulates alginate production through AlgB phosphorylation and AlgW-mediated degradation of MucA. When AlgB is dephosphorylated, AlgU remains sequestered by MucA, allowing expression of acute virulence factors. The FimS/AlgZ-AlgR system contributes to alginate production, twitching motility, and rhamnolipid synthesis. BqsS-BqsR regulates rhamnolipid production, quorum-sensing signals, and biofilm maintenance or dispersion in response to increased Fe²^+^ levels. FleS-FleR also influences biofilm formation and type III secretion system (T3SS) expression, while elevated intracellular c-di-GMP represses FleQ-dependent flagellar gene transcription. CarS-CarR regulates pyocyanin production, protease secretion, alginate synthesis, and swarming motility in response to high Ca²^+^ concentrations. The GacS-GacA system senses environmental signals in CF mucus and activates the response regulator GacA, which induces transcription of the small regulatory RNAs rsmY and rsmZ. This system is represented by RetS when it senses mucin or GlnNac; in contrast, it is activated by LadS when it senses Ca^2+^ in the environment. When RsmY and RsmZ levels are low, the RNA-binding proteins RsmA and RsmN remain active, promoting an acute infection phenotype characterized by enhanced T3SS expression and flagellar and type IV pili motility, while repressing quorum sensing, biofilm formation, exopolysaccharide production, and the type VI secretion system (T6SS). Conversely, high expression of RsmY and RsmZ sequesters RsmA and RsmN, favoring a chronic infection phenotype. Elevated c-di-GMP levels, promoted by diguanylate cyclases (DGCs), are associated with chronic infection, whereas reduced levels mediated by phosphodiesterases (PDEs) correlate with acute infection. PhoR-PhoB regulates pyocyanin production, antibiotic resistance, and biofilm formation and activates the H1-T6SS under low Pi conditions. The PhoP-PhoQ system senses low Mg²^+^, low Ca²^+^, acidic pH, and CAPs, inducing genes involved in lipid A modification. Similarly, low pH, low Mg²^+^, high Fe²^+^, and CAPs activate the PmrA-PmrB system, promoting lipid remodeling. Known ligands are indicated above their corresponding histidine kinases. The figure was created with BioRender.

The establishment of *P. aeruginosa* infection in the CF lung requires an initial crucial step: adherence to a surface. Notably, the CF mucus contains higher amounts of MUC5B and MUC5AC mucins (57–60), which are recognized by type IV pili (fimbriae) and non-pilus adhesins (4, 61–64) of *P. aeruginosa*. After attachment, bacteria begin replicating to form microcolonies and complex structures known as biofilms. Early biofilm development depends on surface attachment mediated by members of the Cup fimbriae family. *P. aeruginosa* encodes *cupA*, *cupB*, and *cupC*, which are regulated by the RocS1-RocA1-RocR TCS (Fig. 1) (65–67). The loci of RocS1, RocR, and RocA1 were identified using transposon mutagenesis (65). In this system, RocA1 activates transcription of *cupB* and *cupC* (65). In contrast, RocR represses Cup fimbriae expression by lowering intracellular c-di-GMP (68), and RocA2 inhibits the expression of the MexAB-OprM efflux pump (Fig. 1). This trait is observed in mature biofilms and in clinical isolates from iwCF. While the ligands for this TCS are unknown, recent pangenome analyzes suggest that the Roc regulon contains additional components, possibly including a fourth response regulator, RocA3, and a novel histidine kinase, RocS4. These findings could expand the regulatory range of the Roc network in *Pseudomonas* species (69). The expression of the type IV pilus (T4P) major subunit, PilA (70), is regulated by the PilS-PilR TCS (71). This T4P is also involved in the attachment of the bacterium to surfaces (Fig. 1) (72). The specific ligands for PilS are also unknown. It is well known that PilS detects high levels of inner-membrane PilA to initiate PilA downregulation (73, 74). Notably, this TCS also activates the FleS-FleR system (Fig. 1) (75). Mutations in *pilS* and *pilR* impair twitching and swimming motility, respectively (75). Overall, the PilS-PilR system is more active during acute infection to promote colonization, but its expression decreases as *P. aeruginosa* enters a biofilm state in chronic infection.

The development of a mucoid phenotype characterizes the transition from acute to chronic infection. *P. aeruginosa* produces alginate to protect itself from the aggressive CF-lung environment. This process is regulated in part by the KinB-AlgB TCS (76). Its functional activation suggests that this TCS is associated with specific growth conditions and environmental contexts of the host. KinB activity has been linked to acute infection conditions. The loss of KinB or inhibition of its phosphatase activity promotes the expression of genes associated with mucoid behavior and chronic adaptation (77). Indeed, *in vivo* infection models have shown that KinB is functionally active during early stages of infection, suggesting that signals from the host and microenvironment influence the regulation of this system (78). In addition, KinB and RpoN regulate AlgW expression. AlgW degrades MucA and releases AlgU (also known as AlgT or σ22), the main regulator of alginate production (Fig. 1) (77, 79, 80). AlgU activates transcription of *algB*, *algR*, and *amrZ*, which, in turn, promotes expression of algD (81, 82). KinB acts as a phosphatase. It keeps AlgB unphosphorylated and prevents AlgW activation, thereby allowing AlgU to bind to MucA. As a result, AlgU/MucA promotes the production of pyocyanin and elastase, but not alginate (Fig. 1) (83). KinB autophosphorylates and transfers the phosphate to AlgB. AlgB then activates AlgW and releases AlgU, which promotes alginate production but not pyocyanin or elastase production (Fig. 1) (83). The ligands sensed by this TCS also remain unknown. Additionally, the FimS-AlgR TCS contributes to alginate production (Fig. 1) and confers a mucoid phenotype (84–86). FimS/AlgZ-AlgR is essential for type IV pili-mediated twitching motility, which is linked to acute infection. However, a downregulation of cAMP-Vfr favors the chronic phenotype (87). AlgR’s regulatory role changes with its phosphorylation: non-phosphorylated AlgR promotes T4P biogenesis. T4P consists of a major pilin (PilA) and several minor pilins, all encoded in the *fimU-pilVWXY1Y2E* operon (85). T4P expression is positively regulated by the virulence factor regulator Vfr and its allosteric effector, cyclic AMP (88). Phosphorylated AlgR activates *algD* and represses the expression of acute virulence factors (84, 89, 90). AlgR induces transcription of *algC* to produce rhamnolipids (Fig. 1) (91, 92).

 *P. aeruginosa* senses the availability of Fe^2+^ in the CF mucus by the BqsS-BqsR TCS (93, 94). This system regulates the dispersion and detachment of mature biofilms. It also regulates the synthesis of two QS signals: N-butyryl-L-homoserine lactone and PQS, and the production of rhamnolipids (95, 96). Additionally, BqsS-BqsR activates genes involved in spermidine biosynthesis and export. This cationic polyamine neutralizes negative membrane charges and protects the bacterium against polymyxins (93). The production of mature biofilm also requires the FleS-FleR TCS, with a significant contribution from FleQ, a master regulator of flagellar genes. FleQ itself relies on signaling from the PilS-PilR TCS (Fig. 1). FleQ is a bacterial enhancer-binding protein in the Ntcr family (97) and is modulated by the second messenger c-di-GMP (98, 99). Higher c-di-GMP promotes FleQ-mediated inhibition of flagellar motility, and higher Ca^2+^ inhibits type III secretion system (T3SS) expression and promotes biofilm formation (100). FleQ directly triggers the transcription of *fleS* and *fleR*, thereby promoting the regulation of flagellar motility and adhesion (Fig. 1) (101, 102). This TCS also regulates AmrZ activity, thereby inducing c-di-GMP synthesis, which tightly controls H1-T6SS expression and activity (103). Under elevated intracellular Ca^2+^, FleR represses ExsA, the positive regulator of the T3SS, independently of FleS (Fig. 1) (104). Thus, FleS-FleR contributes to the transition from acute to chronic infection. The specific ligands sensed by FleS remain unknown.

The availability of Ca^2+^ in the CF mucus is sensed by the CarS-CarR TCS (105), which mediates biofilm formation, pyocyanin production, and swarming and twitching motility (Fig. 1) (105). Together, these processes promote colonization. Higher Ca^2+^ levels induce thicker biofilms, increased *algD* transcription, and enhanced release of the AprA, LasB, and PrpL proteases (106). These changes are accompanied by enhanced alginate production and pyocyanin biosynthesis. The presence of Ca^2+^ is also sensed by LadS (107), the inductor of the GacS-GacA TCS. In this system, GacS is an HK sensor, and GacA is its cognate RR (Fig. 1). This TCS modulates gene expression via the Gac/Rsm signaling pathway (108, 109) and the c-di-GMP pathway (Fig. 1) (110, 111). The system regulates the synthesis of QS signaling molecules, specifically N-acyl homoserine lactones (AHLs) 3O-C12-HSL and C4-HSL, which mediate the synthesis of pyocyanin, hydrogen cyanide, rhamnolipids, and lipase (112). The cooperation between GacS-GacA and AHLs suggests a synergistic role in positively regulating virulence gene expression (113). GacS activity is negatively regulated by RetS when activated by mucin and GlcNAc (114). However, the specific signal that activates GacS remains unknown. Nevertheless, a recent study on the colistin-resistant *P. aeruginosa* strain ST3351 indicates that sublethal colistin doses can induce a switch from a non-mucoid to a mucoid phenotype, suggesting that colistin can act as a ligand for this system (115). This complex regulatory circuit acts as a master switch, modulating the bacteria’s transition from acute to chronic infection (Fig. 1) (108, 116, 117). GacS-GacA controls transcription of two small regulatory RNAs: RsmY and RsmZ (118, 119). These sRNAs bind and sequester the RNA-binding proteins RsmA and RsmN (120), preventing their interaction with target RNAs (Fig. 1). RsmA and RsmN post-transcriptionally regulate expression of virulence-related genes, including those for exopolysaccharide production (Pel, Psl, and alginate) (108), pyoverdine, pyochelin (121), pyocyanin, and elastase production (122, 123), biofilm formation, antimicrobial resistance, motility (117, 121, 124), and T3SS/T6SS function (108, 111). When LadS activates the Gac/Rsm pathway, it increases SadC transcription (125), a diguanylate cyclase (DGC) that synthesizes c-di-GMP. Other DGCs, such as HsbD (126), NicD (127), WspR, and RoeA, also increase. These DGCs elevate c-di-GMP levels, promote biofilm formation, and contribute to infection persistence, increased antibiotic resistance (128), and enhanced T6SS activity. BrlR is a transcriptional regulator that responds to higher c-di-GMP levels. It activates SagS, a TCS that enhances biofilm formation and antibiotic resistance. SagS achieves this by inducing the expression of efflux pumps MexEF-OprN and MexAB-OprM (129). Additionally, AmrZ is a highly conserved transcriptional factor in *P. aeruginosa* and *P. fluorescens*. It influences the transcription of several genes encoding phosphodiesterases involved in c-di-GMP turnover, thereby favoring acute infection (Fig. 1) (111, 130, 131). In *P. aeruginosa*, AmrZ maintains lower levels of c-di-GMP. This results in increased motility, decreased biofilm formation (132), and repression of T3SS activity (111). When the Gac/Rsm system is inactive, RsmY/Z levels are low, and RsmA/N proteins are repressed, thereby repressing transcription of virulence factors associated with the chronic infection phenotype (e.g., alginate production and biofilm development). Additionally, RetS represses GacS-GacA signaling (Fig. 1) and maintains low c-di-GMP levels (111). These lower levels (RsmY/Z) promote motility and T3SS function (Fig. 1), traits typically associated with the acute infection phenotype. When the Gac/Rsm system is active, RsmY/Z levels increase, sequestering RsmA/N and thereby promoting the transcription of virulence factors associated with chronic infection, such as biofilm development and T6SS activity, while downregulating T3SS activity (Fig. 1) (41, 111, 117, 133, 134). These findings suggest that the Gac/Rsm system facilitates the transition of *P. aeruginosa* from an acute to a chronic infection during prolonged colonization.

Furthermore, the lower availability of inorganic phosphate (Pi) in the CF mucus is sensed by the PhoR-PhoB TCS to promote biofilm formation, activity of the T6SS, and production of pyocyanin (135, 136). The Pho regulon in *P. aeruginosa* comprises the *phoBR*, *pstSCAB*, and *phoU* genes (137, 138). The PhoR-PhoB TCS enables the bacterium to sense and maintain Pi homeostasis under Pi limitation (139). PhoR expression is induced through interactions with the high-affinity Pi transporter PstABC via PhoU. Under phosphate-replete conditions, PstABC imports Pi. PhoU promotes the phosphatase activity of PhoR, thereby inactivating the PhoR-PhoB TCS (140). In contrast, this inhibition is relieved under phosphate-limiting conditions (141). PhoR then autophosphorylates and activates PhoB. Activated PhoB induces the transcription of multiple genes associated with bacterial virulence and biofilm formation (Fig. 1) (142, 143). In contrast, *P. aeruginosa* also expresses PitA, a low-affinity Pi transporter. This reduces intracellular Pi levels and induces the expression of H2- and H3-T6SS genes through the PhoB and QS pathways (135). Thus, PhoR-PhoB activation provides a survival advantage by coupling nutrient sensing with adaptive responses characteristic of the chronic infection.

The biofilm is a complex structure composed of an extracellular matrix that contains extracellular DNA (144). This DNA works as a chelator for Mg^2+^ and other divalent ions (55). Thus, this extracellular DNA creates a localized cation-limited environment that activates the PhoP-PhoQ TCS. This TCS is also activated by cationic antimicrobial peptides (CAPs), such as polymyxin B, and by decreased pH (Fig. 1) (145–150). The operon encoding this system consists of *orpH*, *phoP*, and *phoQ*. Once activated, PhoP-PhoQ induces the *pagP* gene, which encodes an outer membrane enzyme that transfers a palmitate residue from phospholipids to lipid A (151). This palmitoylation (hexa-acylated lipid A) enhances resistance to CAPs (152). During chronic infection, a hepta-acylated lipid A is also produced (153). In addition to regulating *pagP* and lipid A modifications, the PhoP-PhoQ system in *P. aeruginosa* functions as a global regulator. A recent study identified novel PhoP*-*regulated genes, including those involved in membrane integrity, transport, and stress response (154). Furthermore, lipid A from *P. aeruginosa* clinical isolates lacking PagL, an enzyme that mediates deacylation (the removal of the 3-OH group from C10 fatty acids), suggests that the loss of PagL is a feature of long-term adaptation to the CF airway (155). Lipid A can also be hydroxylated in secondary acyl chains by the dioxygenases LpxO1 and LpxO2. These structural modifications are associated with resistance to CAPs, *in vivo* persistence, and pathogenicity (156). Indeed, nebulized colistin treatment can select for *P. aeruginosa* variants that enhance lipid A remodeling efficacy (157). Together, these findings suggest that *P. aeruginosa* can sense multiple cues in CF mucus (Fig. 1) (158, 159). In addition, PmrA-PmrB TCS also detects lower pH and low Mg^2+^ concentration (Fig. 1) (158, 160–162). Phosphorylated PmrA activates the operon *arnBCADTEF* and *pmrCAB*, which are responsible for the addition of L-4-aminoarabinose (L-Ara4N) and phosphoethanolamine (pEtN) to lipid A. They also modify the LPS core by adding PEtN via *cptA*, adding L-Ara4N via *pmrE*, and generating deacylated lipid A via *lpxR* (163, 164) (Fig. 1). The PmrA-PmrB system indirectly represses *lpxT* via *pmrR* (165). These changes lower the LPS net negative charge and increase resistance to polymyxins. Chronic colistin treatment in CF patients promotes the *pmrB* gain-of-function. Indeed, a mutation in *pmrB* confers high-level polymyxin resistance and enhances alginate production, thereby contributing to the chronic infection phenotype in the iwCF (166). Mutations in PmrA-PmrB (167, 168) are required for *pmrH* overexpression, which is essential in colistin resistance (167). Some mutations acquired during infection can alter lipid A structure, thereby promoting polymyxin resistance. This system can also be activated by extracellular DNA (14) and Fe^2+^ (169). Increased airway iron availability may help *P. aeruginosa* persist in iwCF.

## CONCLUSIONS

TCSs constitute a key regulatory network in the transition of *P. aeruginosa* from an acute to a chronic infection phenotype in iwCF. However, the evidence discussed in this review reveals significant gaps in our understanding of this adaptation, particularly in identifying physiological ligands (e.g., ions such as Ca²^+^, Fe²^+^, Mg^2+^, Zn^2+^, and Pi, low pH, neutrophil-derived factors, including calprotectin and extracellular DNA) for TCSs, such as PilS-PilR, KinB-AlgB, FleS-FleR, RocS-RocA/RocR, and GacS-GacA. The absence of ligands for some TCSs limits our understanding of how these signals are detected and transduced to reprogram the expression of secretion systems such as T3SS and T6SS, pigments, exopolysaccharides, and lipopolysaccharide remodeling. Likewise, crosstalk between the central TCS and QS systems (Las, Rhl, PQS, and IQS) remains poorly explored, despite their likely role as a regulatory axis in the transition to chronic infection phenotypes. In this context, TCSs such as PmrA-PmrB, PhoP-PhoQ, KinB-AlgB, and GacS-GacA emerge as promising therapeutic candidates, and their study, together with the functional characterization of their ligands, could open important new therapeutic strategies to limit the persistence and antibiotic resistance of *P. aeruginosa* in iwCF and thereby reduce the morbidity and mortality rates in these patients.

Taken together, these observations suggest that signals present in CF mucus, combined with antibiotic pressure, activate TCSs, promoting chronic over acute infections. The sustained signaling drives phenotypic adaptations. Consequently, these adaptations enhance bacterial persistence in the CF lung and, ultimately, contribute to progressive loss of lung function and increased morbidity and mortality in iwCF.

## AFTER EPUB

*[This article was published on 25 February 2026 with incorrect spelling of the author, Vianney Ortiz-Navarrete. The spelling was corrected in the current version, posted on 4 March 2026.]*
